# Machine learning methods to predict cadmium (Cd) concentration in rice grain and support soil management at a regional scale

**DOI:** 10.1016/j.fmre.2023.02.016

**Published:** 2023-03-10

**Authors:** Bo-Yang Huang, Qi-Xin Lü, Zhi-Xian Tang, Zhong Tang, Hong-Ping Chen, Xin-Ping Yang, Fang-Jie Zhao, Peng Wang

**Affiliations:** a Jiangsu Collaborative Innovation Center for Solid Organic Waste Resource Utilization*,* College of Resources and Environmental Sciences, Nanjing Agricultural University*, Nanjing* 210095*,* China; b Centre for Agriculture and Health, Academy for Advanced Interdisciplinary Studies*,* Nanjing Agricultural University*, Nanjing* 210095*,* China

**Keywords:** Cadmium, Food safety, Heave metals, Machine learning, Soil contamination, Predictive model

## Abstract

Rice is a major dietary source of the toxic metal cadmium (Cd). Concentration of Cd in rice grain varies widely at the regional scale, and it is challenging to predict grain Cd concentration using soil properties. The lack of reliable predictive models hampers management of contaminated soils. Here, we conducted a three-year survey of 601 pairs of soil and rice samples at a regional scale. Approximately 78.3% of the soil samples exceeded the soil screening values for Cd in China, and 53.9% of rice grain samples exceeded the Chinese maximum permissible limit for Cd. Predictive models were developed using multiple linear regression and machine learning methods. The correlations between rice grain Cd and soil total Cd concentrations were poor (*R*^2^ < 0.17). Both linear regression and machine learning methods identified four key factors that significantly affect grain Cd concentrations, including Fe-Mn oxide bound Cd, soil pH, field soil moisture content, and the concentration of soil reducible Mn. The machine learning-based support vector machine model showed the best performance (*R*^2^ = 0.87) in predicting grain Cd concentrations at a regional scale, followed by machine learning-based random forest model (*R*^2^ = 0.67), and back propagation neural network model (*R*^2^ = 0.64). Scenario simulations revealed that liming soil to a target pH of 6.5 could be one of the most cost-effective approaches to reduce the exceedance of Cd in rice grain. Taken together, these results show that machine learning methods can be used to predict Cd concentration in rice grain reliably at a regional scale and to support soil management and safe rice production.

## Graphical abstract

**Figure:**
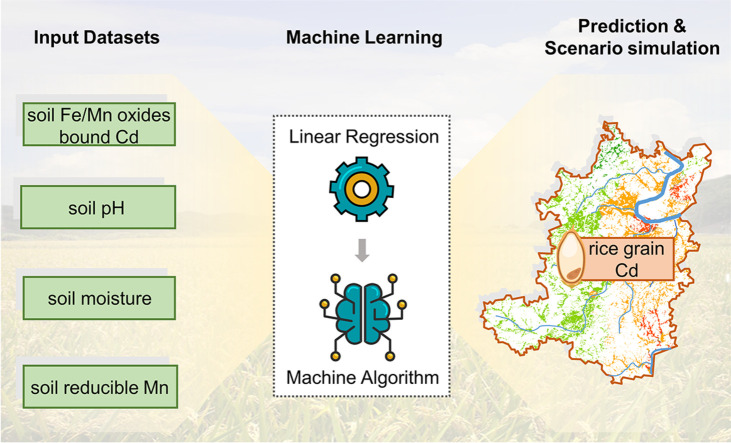

## 1 Introduction

Cadmium (Cd) is a toxic metal that is ubiquitous in agricultural soils. Compared with other toxic metals, Cd is more readily taken up by crops and subsequently accumulated in the edible parts [1,2], making it a common contaminant in foodstuffs [1,^3^,4]. The elevated accumulation of Cd in some food commodities, such as grains of rice (*Oryza sativa*) and wheat (*Triticum aestivum*), and products of cocoa (*Theobroma cacao*), is an issue affecting both human health and international trade [3]. Globally, dietary intake from foods is the primary exposure pathway to the general non-smoking population, with plant-derived foods being the dominant contributors among various foods [1,[4], [5], [6]. For some populations in many Asian countries and subgroups in some European countries, the average dietary intake of Cd exceeds the threshold (10.8 µg/kg body weight/month) recommended by European Food Safety Authority [3,4]. In some regions of southeast Asian countries, dietary Cd intake even exceeds the level of tolerable intake (25 µg/kg body weight/month) established by the Food and Agriculture Organization (FAO) and World Health Organization (WHO) [7,8]. An important reason for the elevated Cd accumulation in food is soil contamination. In China, for example, a nationwide survey revealed that approximately 7% of the agricultural soil samples had Cd concentrations over the soil environmental quality standards for Cd, ranking the highest among all contaminants [9]. Soil acidification also renders Cd more available for plant uptake [10], [11], [12]. To manage contaminated soils for production of safe foods, it is imperative to be able to predict Cd concentration in the edible parts of food crops using soil properties.

For upland crops such as wheat, a satisfactory multiple linear regression model (*R^2^* = 0.53) has been developed to predict the accumulation of Cd in wheat grain using soil properties, with soil total Cd concentration and pH being the significant influencing factors [13]. In contrast, multiple linear regression models are much less satisfactory for predicting grain Cd accumulation in rice at large regional scales, and the correlations between rice grain Cd and soil Cd concentrations are generally poor (*R^2^* < 0.2) [8,14]. The poor correlations suggest that soil Cd concentration is not a good indicator for predicting grain Cd accumulation in rice. Furthermore, the inclusions of soil pH, organic matter (OM), and other soil properties do not improve the fitting performance [8]. The lack of a reliable predictive model has caused significant uncertainties in the classification of soil quality and the risk assessment of rice grain Cd accumulation. Therefore, it is not surprising that a large proportion of paddy fields with soil Cd concentrations exceeding the soil risk screening values (i.e., classified as ‘contaminated’ soils) produce rice grain with Cd concentrations below the food limit. On the contrary, grain Cd concentrations can exceed the food limit even when soil Cd concentrations are below the risk screening values (i.e., the so-called ‘uncontaminated’ soils). The poor relationships between rice grain Cd and soil Cd concentrations [8,14] suggest that key factors influencing grain Cd accumulation have not been considered or that the linear regression-based methods are inappropriate.

Machine learning provides algorithms to mine hidden patterns or infer correlations from cluttered, irregular, and high-dimensional data, combining the main functions of classification and regression [15,16]. To date, machine learning has been used in a wide range of research fields, including prediction of toxicity of substances in water and soils [17], [18], [19], source identification and environmental behavior modeling of pollutants [20,21], receptor identification of endocrine disruption [22,23], and prediction of air particulate matter content [24,25]. Generally, machine learning consists of three pivotal procedures, namely, the input of training data (feature), construction of the subject model, and output of target results (label) [16,26]. Once the training model passes the validation, the corresponding output can be quickly predicted after taking the new input. Whether machine learning methods can be used to predict Cd concentrations in rice grain at regional scales remains to be investigated.

In the present study, we developed machine learning methods to predict grain Cd concentrations at a regional scale by incorporating key factors that impact soil Cd availability and Cd uptake by rice plants. The machine learning-based methods were also compared with the multiple linear regression model. Finally, based on the reliable machine learning method, we conducted scenario analyses to inform soil management and regulations of Cd-contaminated soils for safe production of rice.

## 2 Materials and methods

### 2.1 The study area

**Fig. 1:**
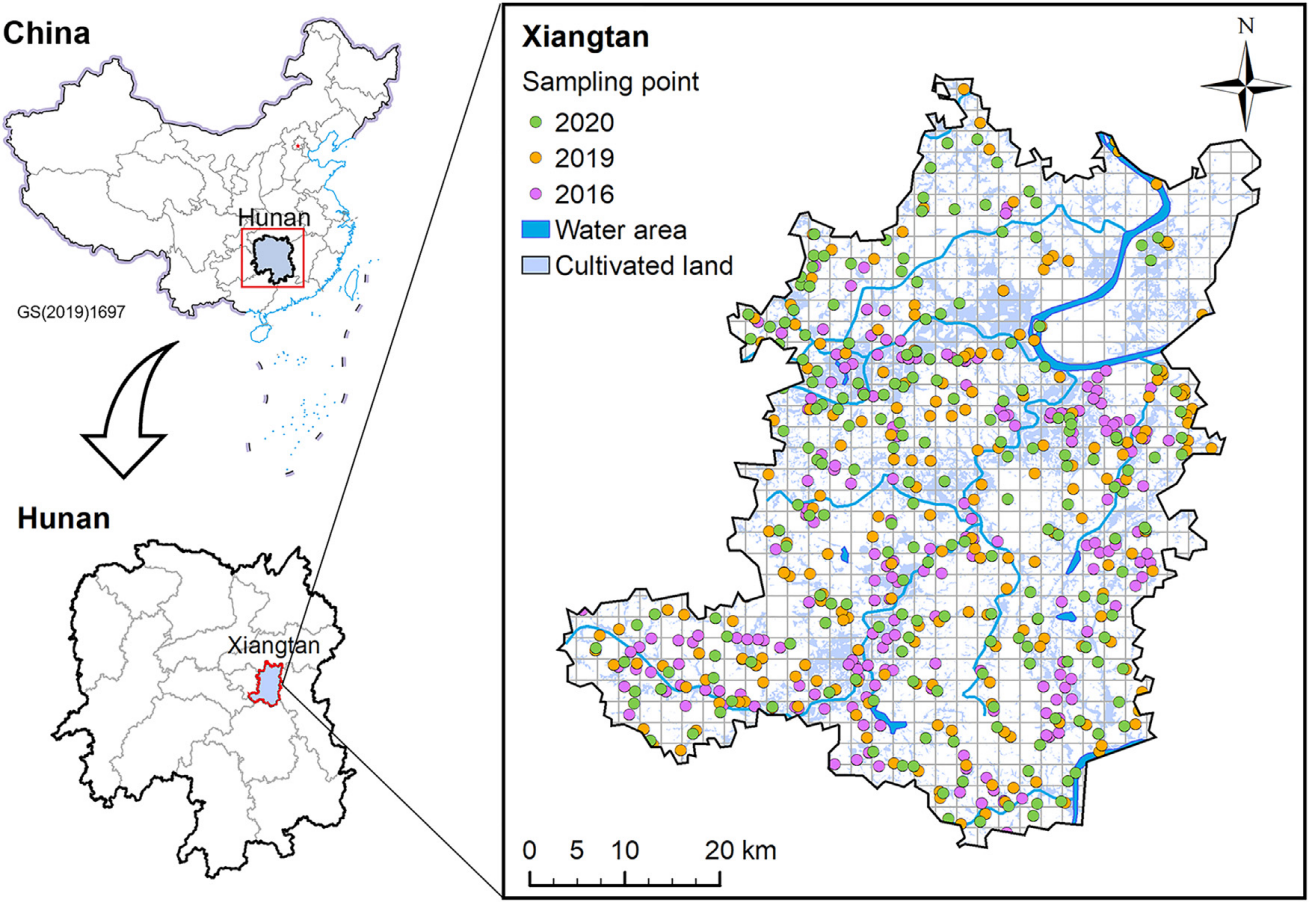
**The study area and sampling sites of paired soil and rice grain samples in 2016 (n =200 pairs), 2019 (n =201 pairs), and 2020 (n =200 pairs).** Map of China is edited on Chinese standard map GS(2019)1697.

The study area is located at Xiangtan, Hunan Province, Southern China (Fig. 1). This area covers ca. 2,791 km^2^, with 842.9 km^2^ being used for farmlands, and is a major rice production region in Hunan Province. Among the farmlands, more than 80% are used for growing rice with irrigation from the Hsiang River and the adjacent reservoirs [27]. The climate is subtropical eastern humid monsoon with an annual mean temperature of 17.5 °C and an annual precipitation of 1,350 mm. The study area has a wide variety of soil types, including Ferrosols-Udic, Ferrosols-Ustic, and Argosols-Udic [28].

### 2.2 Paired soil and rice grain sampling

Paired soil and rice grain samples were collected about a week before rice harvest from Sept. to Oct. in 2016 (n = 200 pairs), 2019 (n = 201 pairs), and 2020 (n = 200 pairs). The sampling sites were relatively evenly distributed across the region (Fig. 1). Each soil and rice grain sample consisted of five subsamples evenly distributed within a paddy field. At each sampling site, ca. 500 g of soil samples were bulked from the top 20 cm soil layer, and ca. 200 g of rice grain samples were collected from the field. Rice grain samples were oven-dried at 60 °C for 48 h before being de-husked for analysis of Cd concentrations.

In 2016, all soil samples were air-dried before analyses of soil properties and soil total Cd concentration, with details presented in a previous study [8]. In that study, we found a poor correlation between rice grain Cd and soil Cd concentrations, and the incorporation of soil properties such as pH and OM content did not significantly improve the correlation. In both 2019 and 2020, therefore, we sampled paired field-moist soil and rice grain samples from the same region, with a particular focus on the analyses of field-moist soils. The field-moist soil samples were bulked from the five subsamples of soils from the top 20 cm soil layer, and soil moisture content at this point could represent the average soil water status during the late grain filling period. One subsample of field-moist soils was tightly sealed on site in 50 mL polypropylene tubes for measurements of field soil moisture content, soil pH, and soil extractions (see below). The other subsample of field-moist soils was air-dried and sieved < 2 mm for soil pH measurement and soil extractions, or < 0.15 mm for analyzing soil total metal concentrations.

### 2.3 Chemical analysis

For field-moist soils, the moisture content was measured by a thermogravimetric method [29]. The pH value of both field-moist and air-dried soils was determined in a 1:2.5 (w/v) soil-water suspension using a glass pH electrode [29]. Total organic carbon (TOC) was determined using a TOC analyzer (Analytik Jena, multi N/C, Germany).

For extractions of both field-moist and air-dried soils, soil samples were extracted with either 0.01 M CaCl_2_ (denoted hereafter as ‘extractable Cd’, representing exchangeable Cd as described previously [30]) or 1.0 M NH_2_OH·HCl (denoted as ‘amorphous Fe-Mn oxides-bound Cd’, reflecting the Cd fraction that can be potentially released to soil porewater during soil drainage [31]). In brief, ca. 5.0 g soil (on a dry weight basis) was weighed into a 50 mL polypropylene centrifuge tube, mixed with 25 mL of extractant, and shaken end-over-end for 4 h before being centrifuged at 4,000 g for 5 min. The supernatant was filtered (0.45 µm) and acidified with 70% concentrated HNO_3_ before analysis. The concentrations of Cd, Mn, and other metals in the extracts were determined by inductively coupled plasma mass spectrometry (ICP-MS, Perkin Elmer NexION 300, USA).

To determine soil total metal concentration, air-dried soil was digested with aqua regia (80/20 HCl/HNO_3_, v/v) in a heating block [32]. Grain samples were digested with high-purity concentrated HNO_3_ with a microwave digester (CEM, Mars, USA) [33]. All digests were diluted with 2% HNO_3_ and filtered (0.45 µm) before analysis by ICP-MS. Quality control measures included the addition of indium (In) as the internal standard, use of procedural blanks and duplicates, and repeated analysis of certified reference materials (including GBW07428 for soil and GBW10045a for rice). Repeated analysis of the two certified references yielded mean Cd concentrations of 0.22 ± 0.01 mg/kg (n = 6) for GBW07428 and 0.34 ± 0.01 mg/kg (n = 6) for GBW10045a. These values were in good agreement with their certified values of 0.20 ± 0.02 mg/kg and 0.32 ± 0.04 mg/kg, respectively.

### 2.4 Modeling for predicting grain Cd concentrations

Multiple linear regression and machine learning methods were used to establish models to predict Cd concentrations in rice grain using soil properties. Soil properties included pH, moisture content of field-moist soil, and concentrations of total and chemically extracted metals in both field-moist and air-dried soils.

In multiple linear regression, the best prediction was obtained using ordinary least square and stepwise regression with an assumption of linear relationships between dependent and independent variables [34]. Where appropriate, variables that showed skewed distributions were logarithmically transformed before statistical testing or modeling to achieve normality and homogeneity of variances.

In machine learning, we compared three methods, namely, back propagation neural network (BP-NN), random forest (RF), and support vector machine (SVM). BP-NN, a multilayer feedforward network, is trained by error back propagation algorithm [35]. The three machine learning methods are used because they are not limited to a simple linear or polynomial relationship and rely less on prior experience [36]. Furthermore, the performance of the three methods can be continuously improved as more data are obtained [15]. The basic learning rule of BP-NN is to use back propagation to regulate the weight and threshold value of the network to gain the minimum error sum of squares according to the steepest descent method [35,37]. RF, a non-parametric and nonlinear method, combines ensemble learning with the decision tree [38]. RF builds multiple independent decision trees based on bootstrap sampling and determines the optimal prediction by averaging the regression result of all trees [38,39]. SVM, a kernel-based supervised technique, has significant generalization and convergence performance [40]. The training algorithm of SVM is to find a new partition hyperplane by projecting data into a high- or infinite-dimensional space to simplify complex nonlinear relationships [41,42].

Because the 2016 survey did not have field-moist soil data, data from the 2019 and 2020 surveys (n = 401) were pooled for subsequent model development. During the machine learning analyses, we randomly divided the 401 paired datasets into 280 training sets and 121 validation sets (a ratio of 7/3) using the *sample* function in R 4.1.1 [43]. The data distribution and statistics of training and validation datasets are presented in Table S1. The functions and optimized parameters of these three methods are presented in Table S2. In addition, the coefficients of determination (*R*^2^), root mean squared error (RMSE), and bias were compared to assess the accuracies of each model.

### 2.5 Statistical analysis

Geographic information maps were drawn using ArcGIS 10.5 (Esri, Inc, USA), and Ordinary Kriging was used for the spatial interpolation. SPSS 25 (IBM, USA) was used for descriptive statistics, Kolmogorov-Smirnov test, Friedman test, ANOVA, correlation, and regression analysis. The BP-NN, RF, and SVM methods were built using the *neuralnet, randomForest*, and *e1071* packages in the R program (version 4.1.1), respectively. The *e1071* package was also used for the ten-fold cross-validation of machine learning models. Scenario simulations were performed based on the optimal predictive model (i.e., the SVM method).

## 3 Results

### 3.1 Paired soil and rice samples

**Fig. 2:**
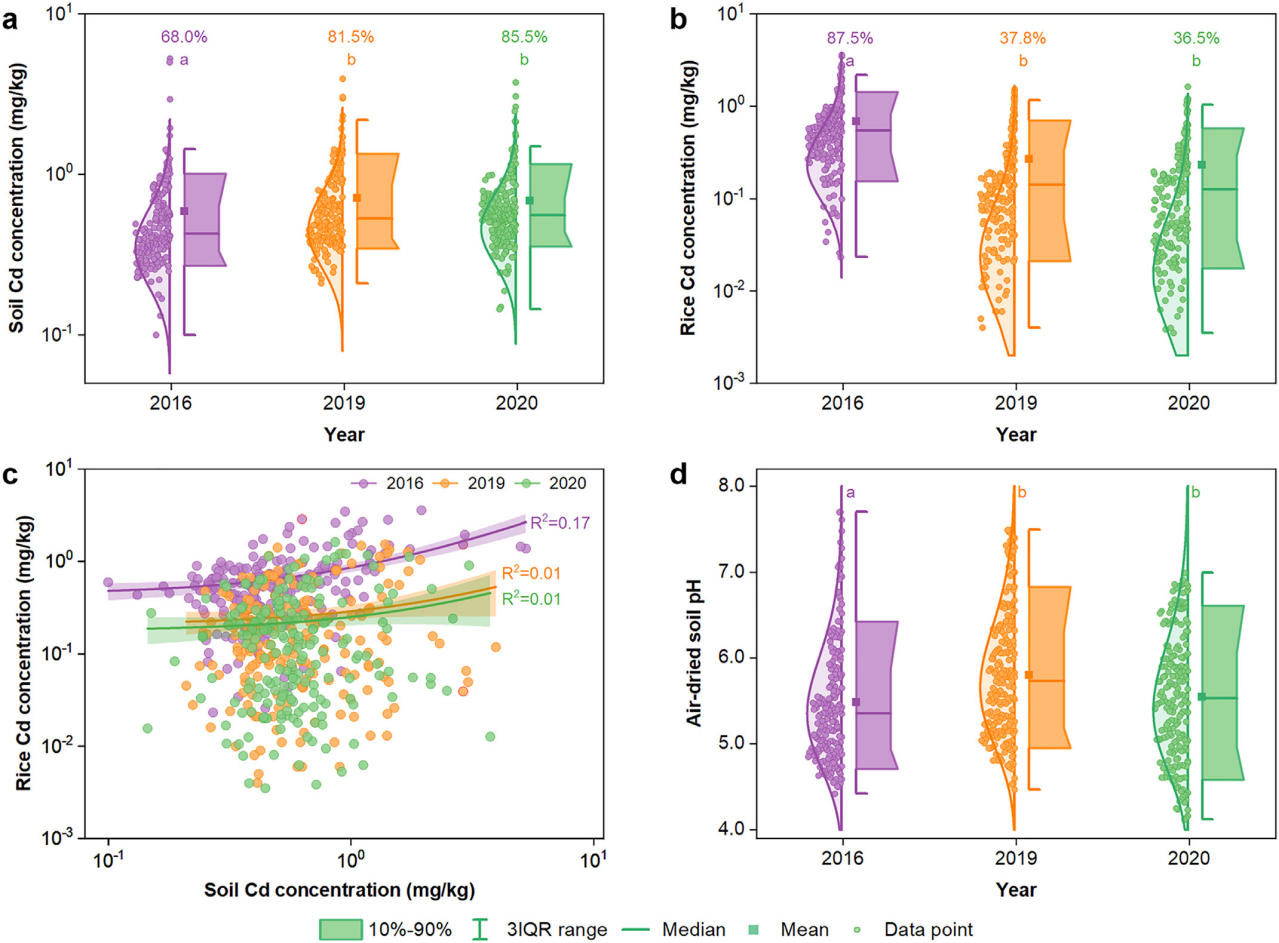
**Soil Cd concentrations (a), rice grain Cd concentrations (b), the correlations between soil Cd and rice Cd concentrations (c), and soil pH values (d) in the 2016, 2019 and 2020 surveys.** The numbers in (a) and (b) are exceedance rates of Cd in soil and rice samples. Different letters represent significant differences in the median values between different years at a level of 0.05.

Soils collected from the three harvest years varied widely in pH (4.12 – 7.70), TOC (2.74 – 52.0 g/kg), total Cd (0.10 – 5.26 mg/kg), total Mn (71.8 – 2655 mg/kg), total Zn (54.6 – 956 mg/kg), and total Cu (16.7 – 112 mg/kg) (Table S3). Soil Cd concentrations in the 2016 survey varied from 0.10 to 5.26 mg/kg (mean 0.60 mg/kg, median 0.43 mg/kg) (Fig. 2a), with 68.0% of the soil samples having Cd concentrations exceeding the Chinese soil risk screening values (i.e., 0.3 mg/kg when pH < 5.5, 0.4 mg/kg when 5.5 ≤ pH < 6.5, 0.6 mg/kg when 6.5 < pH ≤ 7.5, and 0.8 mg/kg when pH > 7.5; GB15618-2018) [44]. Soil Cd concentrations ranged from 0.21 to 3.96 mg/kg (mean 0.72 mg/kg, median 0.54 mg/kg) in the 2019 survey and from 0.15 to 3.75 mg/kg (mean 0.68 mg/kg, median 0.55 mg/kg) in the 2020 survey (Fig. 2a). The exceedance rates were 81.5% and 85.5% in the 2019 and 2020 surveys, respectively. Approximately 2.5%, 3.5%, and 4.5% of the soil samples had Cd concentrations above the Chinese risk intervention values (i.e., 1.5 mg/kg when pH < 5.5, 2.0 mg/kg when 5.5 ≤ pH < 6.5, 3.0 mg/kg when 6.5 < pH ≤ 7.5, and 4.0 mg/kg when pH > 7.5; GB15618-2018) in 2016, 2019, and 2020 surveys, respectively. These results indicate widespread soil contamination with Cd in the study area. Soil Cd concentrations showed a large spatial variation; high Cd concentrations were found in the northeast region near the Hsiang River, where intensive industrial activities exist (Fig. S1a-c). The mean soil Cd concentration in the 2016 survey was lower than those of the last two surveys, likely due to fewer sampling sites in the northeast region in the 2016 survey (Fig. 1).

The concentrations of Cd in rice grains varied by three orders of magnitude, ranging from 0.02 to 3.61 mg/kg (mean 0.69 mg/kg, median 0.54 mg/kg) in the 2016 survey, from 0.004 to 1.53 mg/kg (mean 0.27 mg/kg, median 0.14 mg/kg) in the 2019 survey, and from 0.003 to 1.63 mg/kg (mean 0.23 mg/kg, median 0.13 mg/kg) in the 2020 survey (Fig. 2b). According to the Chinese maximum permissible limit of Cd for rice (0.2 mg/kg) (GB2762-2022) [45], the percentages of rice grain samples with Cd concentrations exceeding this limit were 87.5%, 37.8%, and 36.5% in 2016, 2019, and 2020 surveys, respectively, suggesting a considerable decrease in the exceedance in the 2019 and 2020 surveys compared to the 2016 survey. High concentrations of grain Cd were mainly found in the northeast region, a spatial pattern similar to soil Cd concentration (Fig. S1d-f).

The correlations between rice grain Cd concentration and soil total Cd concentrations were poor, with the *R*^2^ value being 0.17, 0.01, and 0.01 for 2016, 2019, and 2020 surveys, respectively (Fig. 2c). According to the Chinese soil environment quality standard (GB15618-2018) and the rice grain Cd limit (GB2762-2022), there were self-contradictory situations; a large proportion of field sites that had a soil Cd concentration exceeding the soil risk screening value produced rice grain with a Cd concentration below the rice safety limit; these represent the false positive cases and account for 7.5%, 46.7%, and 52.0% of all sites in 2016, 2019, and 2020, respectively (Fig. S2). On the contrary, 21.0%, 3.0%, and 3.5% of the field sites with a soil Cd concentration below the screening values had a grain Cd concentration exceeding the rice safety limit in 2016, 2019, and 2020, respectively; these represent the false negative cases (Fig. S2). The total misjudgement rates were 28.5%, 49.7%, and 55.5% in 2016, 2019, and 2020 surveys, indicating substantial uncertainties in the classification of soil quality.

pH in air-dried soil ranged from 4.42 to 7.70 (mean 5.48, median 5.36) in the 2016 survey, while the median pH significantly increased by 0.36 units in the 2019 survey (ranging from 4.47 to 7.49; mean 5.79, median 5.72) and by 0.27 units in the 2020 survey (ranging from 4.12 to 6.99; mean 5.55, median 5.53) (Fig. 2d). The median pH values in the 2019 and 2020 surveys were not significantly different.

### 3.2 Linear regression model to predict rice grain Cd concentration

As shown in Fig. 2c, soil Cd concentration correlates poorly with grain Cd concentration. Therefore, multiple linear regression analysis was used to fit a predictive model for rice grain Cd concentration. Variables included total and extractable Cd concentrations, as well as properties of field-moist and air-dried soils. Of all soil variables, soil Cd concentration ([C_t-Cd_]), extractable Cd ([C_e-Cd_]), amorphous Fe-Mn oxides-bound Cd ([C_Fe/Mn-Cd_]), reducible Mn concentration ([C_Fe/Mn-Mn_], extracted by NH_2_OH·HCl [46,47]), air-dried pH ([pH_air-dried_]), and field-soil moisture content ([MC]) were found to be significant variables in the multiple linear regression analysis (Table S4). Soil total Cd, extractable Cd, and amorphous Fe-Mn oxides-bound Cd alone explained 0.3%, 24.1%, and 29.3% of the variance in grain Cd concentration, respectively. Incorporating soil pH in the regression model improved the coefficient of determination by 10% – 13% (Table S4). Similarly, inclusions of soil-reducible Mn concentration and moisture content further improved the fitting by 0.7% – 6% and 4% – 10%, respectively. The combination of soil amorphous Fe-Mn oxides-bound Cd, reducible Mn concentration, air-dried soil pH, and soil moisture yielded the best regression model, which explained 56.3% of the variance in grain Cd concentration with a RMSE value of 0.39 (Table S4 and Fig. S3).

### 3.3 Machine learning models to predict rice grain Cd concentration

**Fig. 3:**
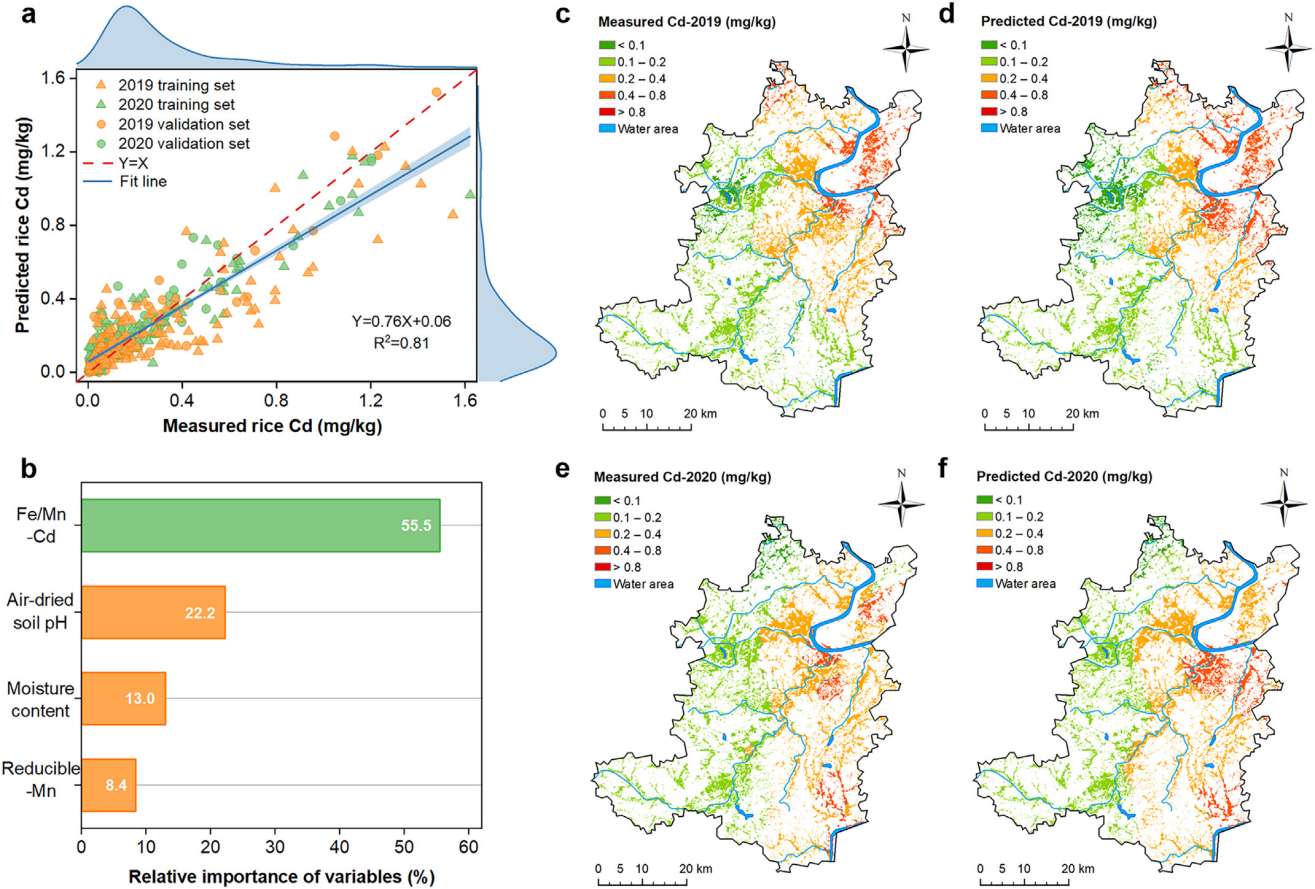
**Prediction of rice grain Cd concentrations in 2019 and 2020 surveys based on the machine learning-based support vector machine (SVM) model.** (a) Scatter plots of measured grain Cd concentrations versus predicted values (n = 401), consisting of 280 training datasets and 121 test datasets. (b) The relative importance of variables in the SVM model, including soil amorphous Fe-Mn oxides-bound Cd (Fe/Mn-Cd), air-dried soil pH, soil moisture content, and reducible Mn concentration. Spatial distribution maps of measured and predicted grain Cd concentrations in 2019 (c, d) and 2020 (e, f).

Because the relationships between rice grain Cd concentrations and soil variables are not always linear, we used three machine learning methods (BP-NN, RF, and SVM) to fit a model to predict rice grain Cd concentration. Consistent with the multiple linear regression model, four variables (amorphous Fe-Mn oxides-bound Cd, reducible Mn concentration, air-dried soil pH, and soil moisture content) were identified as significant factors in the three machine learning-based methods. The *R*^2^ values for the validation sets were 0.64, 0.67, and 0.87 for the BP-NN, RF, and SVM methods, respectively, with the RMSE values being 0.18, 0.17, and 0.11 and the bias being 0.02, 0.01, and 0.02, respectively (Fig. S4), suggesting that SVM is the best method in the fitting performance. When the 401 paired datasets were combined (consisting of 280 training sets and 121 validation sets), the SVM performed the best, with a *R*^2^ of 0.81 (Fig. 3a).

We used the SVM method to evaluate the weights of variables and conduct scenario analysis. Among the four variables, amorphous Fe-Mn oxides-bound Cd is the most important variable that influences grain Cd concentration with a relative weight of 55.5%, followed by air-dried soil pH (22.2%), soil moisture content (13.0%), and soil reducible Mn concentration (8.4%) (Fig. 3b). Using the Ordinary Kriging spatial interpolation, the spatial distribution of rice grain Cd in both 2019 and 2020 surveys were well predicted based on the SVM method (Fig. 3c-f). Furthermore, in the SVM method using the four soil variables as indicators, the total misjudgment rates were reduced substantially to 19.4% and 12.0% in the 2019 and 2020 surveys, respectively, compared with 49.7% and 55.5% in the two respective surveys based on the soil quality standard and the food limit.

### 3.4 Scenario analysis

**Fig. 4:**
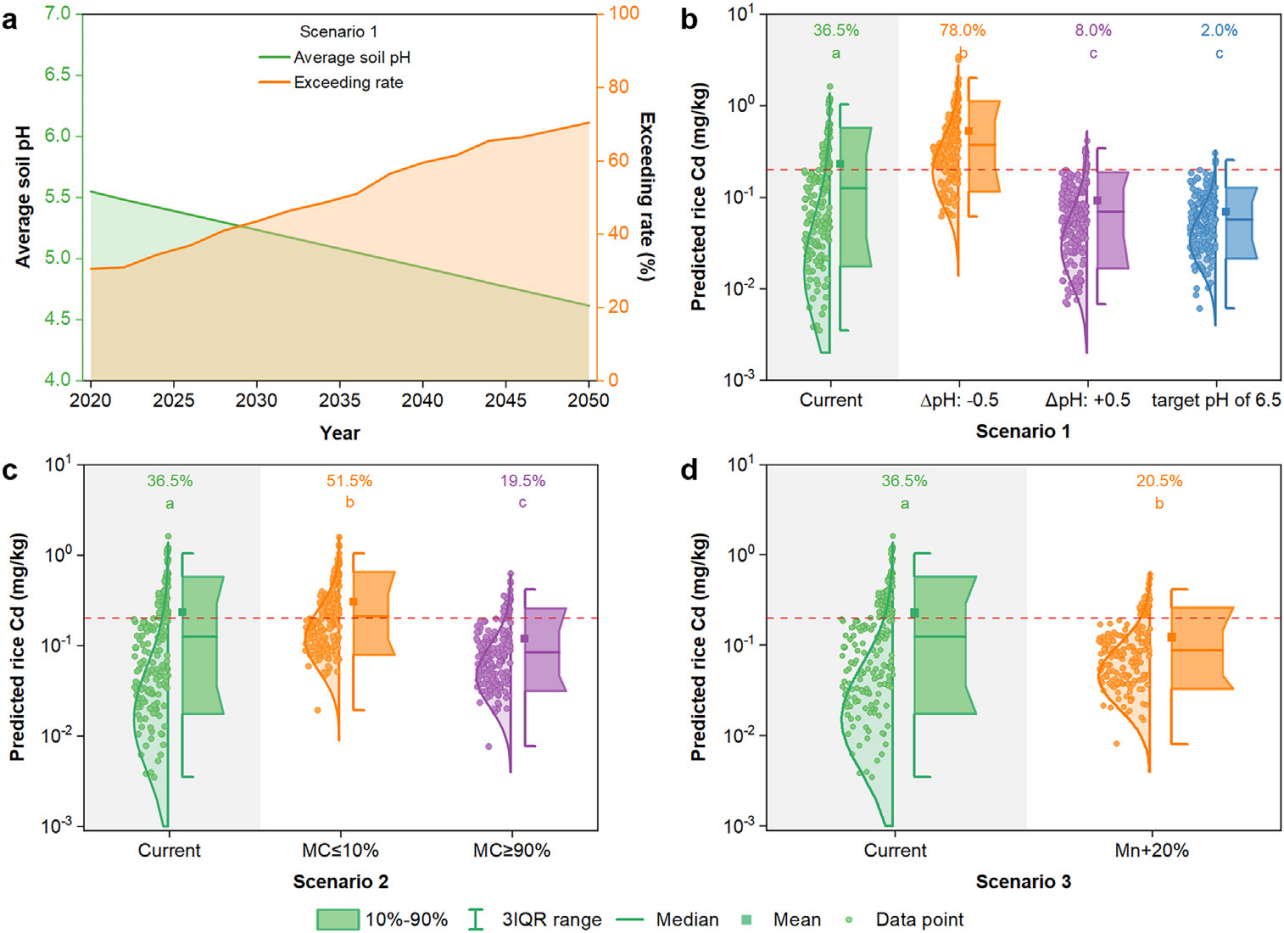
**Scenario simulations based on the SVM model.** (a) The acidification trend of soils and the exceedance rate of rice grain Cd concentration in the upcoming 30 years. (b) Predicted grain Cd concentrations under scenarios of further soil acidification (∆pH: -0.5 units), liming to increase soil pH by 0.5 units (∆pH: +0.5 units), or liming to a target pH of 6.5. (c) Predicted grain Cd concentrations under scenarios of dry or wet climatic conditions, with the dry and wet climate being assumed at 10% and 90% percentiles of the soil moisture frequency distribution, respectively. (d) Predicted grain Cd concentrations under the scenario of increasing concentration of soil reducible Mn by 20%. The dashed lines in (b-d) represent the Chinese maximum permissible limit of Cd for rice (0.2 mg/kg). Different letters represent significant differences in the median values between different scenarios at a level of 0.05.

**Fig. 5:**
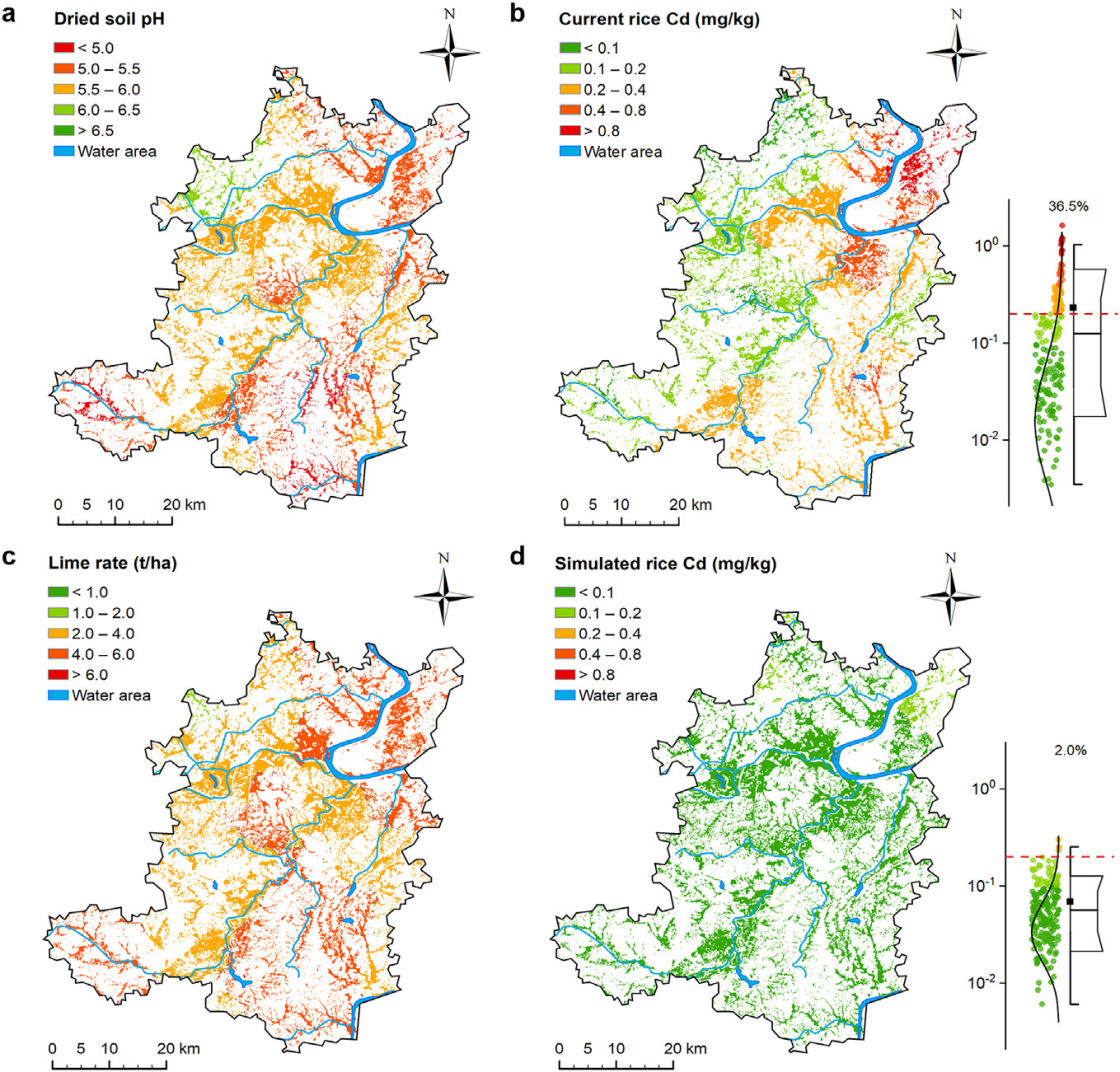
**Scenario simulation of liming with a target soil pH of 6.5.** (a) The current soil pH, and (b) the current rice grain Cd concentrations and exceeding rate. (c) The lime amounts required to adjust soil pH to a target pH of 6.5. (d) Predicted rice grain Cd concentrations under the scenario of liming to increase soil pH to 6.5 based on the SVM model. The dashed lines in (b) and (d) are the Chinese maximum permissible limit of Cd for rice (0.2 mg/kg).

Among the four variables that have significant impacts on grain Cd concentration, soil pH, soil moisture content, and concentration of soil reducible Mn are more easily amenable by soil and agronomic management. We used the SVM model to simulate different scenarios based on the changes in these three variables (Fig. 4). The first scenario is to change soil pH. It has been reported that the median pH of the topsoil in the study area decreased from 6.24 to 5.30 from the 1980s to 2014, with a decline rate of 0.03 units per year [11]. If the soil acidification continues at this rate, it is expected that soil pH value in the study area will decline by 0.5 units in the next 15 years (i.e., around 2035) (Fig. 4a). The corresponding exceedance of rice grain Cd is predicted to double from the current 36.5% to 78.0%. Numerous studies have shown that liming is effective at increasing soil pH value, thereby reducing rice grain Cd concentration [11,^48^,49]. A pH of 6.5 is considered to be the desirable and economic target in terms of effectiveness in lowering rice grain Cd concentration [50]. The scenario simulation shows that raising soil pH by 0.5 units will markedly decrease grain Cd concentrations and decrease the exceedance of grain Cd concentration from 36.5% to 8.0%. To increase soil pH from below 6.5 to the target value of 6.5, 4.13–11.6 t/ha of ground limestone (CaCO_3_) would be required according to a lime model [50], with a total amount of 299,000 t for the entire area under this study (Fig. 5a,c). In this scenario, the exceedance of rice grain Cd will decrease to 2% (Figs. 4b and 5b,d).

Soil moisture content is another variable that negatively affects rice grain Cd concentration (Fig. 3b). Soil moisture is influenced primarily by weather (rainfall) and the timing of paddy water drainage by farmers. In the second scenario, we assessed how changes in the moisture content of field soil impact grain Cd concentration by assuming two extremely dry and wet conditions where the soil moisture content at the time of rice harvest is at 10% and 90% percentiles of the soil moisture frequency distribution, respectively (Fig. 4c). If the study area encounters a dry climate during the rice grain filling period, the exceedance of rice grain Cd is expected to increase from 36.5% to 51.5%. In contrast, if the fields are maintained at wet conditions, the exceedance of rice grain Cd would decrease to 19.5% (Fig. 4c).

Soil reducible Mn concentration has a negative impact on rice grain Cd concentration (Fig. 3b). The third scenario tested is the application of Mn fertilizer. An increase in soil reducible Mn concentration by 20% through applications of Mn fertilizers is expected to reduce the exceedance of grain Cd concentration from 36.5% to 20.5%; this effect is similar in scale to the soil moisture scenario but much smaller than the scenario of liming (Fig. 4d).

## 4 Discussion

### 4.1 Cadmium contamination

The paired soil and rice grain surveys show that Cd contamination is widespread and severe in the study area (Fig. 2). This contamination status has been reported previously in the surveys conducted in the same region and the nearby areas [51], [52], [53]. For example, in a paired soil and rice grain survey (n = 39,642) conducted in 2014 across Changsha, Zhuzhou, and Xiangtan cities (Chang-Zhu-Tan District), Hunan Province, Zhu et al. reported a similar range of soil Cd concentration (from 0.005 to 4.80 mg/kg) and 76% of the rice grain samples exceeding the food Cd limit (mean grain Cd 0.43 mg/kg, median 0.34 mg/kg) [11]. Similarly, Yang et al. surveyed 2,950 paired soil-rice grain samples across Hunan Province in 2015 [54]. They found that 75% of the grain samples exceeded the food Cd limit, with a mean of 0.38 mg/kg and a median of 0.31 mg/kg. There are several reasons responsible for the high exceedance of grain Cd in the region, including soil contamination, soil acidification, and growing of rice cultivars prone to accumulating Cd [10], [11], [12]. The primary source of soil Cd in the study area has been attributed to geogenic weathering and anthropogenic activities [55]. It has been reported that black rock series are distributed in this area, and black shale contains a variety of toxic elements including Cd [56]. The region under study has a long history of metal mining and smelting, causing metal contamination in agricultural soils via both irrigation water and atmospheric deposition [10,^57^,58]. Because the background level of Cd in the soils in Hunan province is not elevated (0.08 mg kg^−1^) [59], anthropogenic activities are likely the major cause of Cd contamination.

Although the mean concentration of soil Cd in the 2006 survey was lower than those in the 2019 and 2020 surveys, the exceedance rate in rice grain Cd in the 2016 survey (87%) was much higher than those (36%–38%) in 2019 and 2020 surveys (Fig. 2b). The large decrease in the exceedance is a combined consequence of the increased soil pH (Fig. 2d) and the introduction of low-Cd rice cultivars in the study area [60]. Since the enforcement of the Action Plan on Soil Pollution and Control (‘Soil Action Plan’) in 2016 [61], the local government has encouraged farmers to lime the soils and grow rice cultivars with low-Cd accumulating ability. Indeed, the median soil pH increased by 0.3–0.4 units from 2016 to 2019/2020 in the study area (Fig. 2d). Based on the SVM predictive model, it is estimated that an increase in soil pH by 0.3–0.4 units would reduce grain Cd concentration by 20%–27% and decrease the exceedance rate by 16%–21%. The magnitude of soil pH increase cannot fully explain the large decrease in the exceedance from 2016 to 2019/2020. Growing low-Cd accumulating rice cultivars is likely another important reason. There are large variations in grain Cd concentrations among rice cultivars [62], [63], [64]. For example, a 10 – 32 fold variation in grain Cd concentration was reported in the 471 locally-adapted and high-yielding rice cultivars when grown at moderately contaminated sites in the study area [64].

### 4.2 Predictive models of rice grain Cd concentration

Consistent with previous studies [8,^14^,^30^,49], rice grain Cd concentrations correlated poorly with soil Cd concentrations in the present study (Fig. 2c). Numerous studies have attempted to develop multiple regression models to predict rice grain Cd concentrations. Generally, this type of model performs well at a small scale, such as a valley [65] or in well-managed paddy fields of the same soil type [66,67], but often fails when applied to a larger scale. For example, a multiple linear regression model incorporating extractable Cd (by 0.1 M CaCl_2_ or 5 mM DTPA), soil pH, and SOM, performed poorly at a large scale, such as across a county (2,130 km^2^) of Hunan Province (*R*^2^ = 0.17, n = 200) [8] and Western Thailand (1,200 km^2^) (*R*^2^ = 0.16, n = 308) [14]. In the present study, the multiple linear regression model incorporating extractable Cd and soil pH explained only 24.4% of the variance in grain Cd concentration (Table S4).

The poor performance of multiple linear regression models at large scales is likely due to the negligence of several key factors. First, soil water content has an important impact on soil Cd availability. Fluctuation in the paddy water status during the grain filling stage can markedly affect grain Cd concentration [68,69]. A typical example displaying the importance of paddy water status is that extractable Cd concentrations correlated closely with pH in air-dried soils (*R*^2^ = 0.75), but the correlation was poor in field moist soils (*R*^2^ = 0.08) (Fig. S5). Soil moisture content is a stochastic variable, depending to a large extent on weather and the timing of soil drainage during the farmer's management. The inclusion of field soil moisture increased the coefficient of determination by approximately 10% (Table S4). Second, uptake of Cd by rice roots is mediated mainly by the Mn transporter OsNRAMP5 (natural resistance-associated macrophage protein 5) [70,71] and to a less extent another Mn transporter OsNRAMP1 [72]. The supply of Mn^2+^ to the root growing medium had a strong inhibitory effect on Cd uptake by rice plants through competition [70,73]. Our results indicate that soil reducible Mn concentration had a negative impact on rice grain Cd concentration and the inclusion of this variable also increased the model fitting performance by 14% (Table S4). Multi-site field trials have shown that soil extractable Mn is an important variable affecting grain Cd concentration with weights of 8%–18% and Mn-containing soil amendments are effective at decreasing grain Cd concentrations [74]. Third, it has been shown that Fe-Mn oxides are the main sorption sites of Cd^2+^ for release into paddy porewater, and the release process is also dependent on soil pH [31]. An increase in Fe-Mn oxide bound Cd resulted in a decreased exchangeable fraction of Cd in paddy soils [75]. Therefore, Fe-Mn oxide bound Cd and soil pH are important variables affecting Cd solubility and gain Cd accumulation, explaining 29% and 14% of the variation in grain Cd concentration, respectively (Table S4). The multiple linear regression model incorporating these variables explains 56.3% of the variance at a county scale (Table S4).

Apart from the linear regression model, we found that the machine learning-based SVM method substantially improved the accuracy of the prediction of grain Cd concentrations (*R*^2^ = 0.81) (Fig. 3a). The remaining 19% uncertainty may be attributable to the different rice cultivars grown in the study area and random errors. Linear regression cannot function well when nonlinear relationships exist between the soil properties and predictors [76]. As shown in the present study, the relationship between amorphous Fe-Mn oxides-bound Cd and soil pH is nonlinear (Fig. S5). This may explain the poor performance of the multiple linear regression model compared with the machine learning models. Moreover, machine learning has the benefits in solving the non-stationarity phenomena compared to multiple linear regression [77,78]. Among the three machine learning methods used in the present study, the SVM has a remarkable generalization performance. Instead of requiring nonlinear optimization with the danger of getting stuck in local minima when using the BP-NN method, the SVM method can solve a linearly constrained quadratic programming problem [79]. Furthermore, in the case of four variables, the SVM method can avoid the problem of poor initial fitting performance better than the RF method [26]. Therefore, the SVM algorithm is more reliable in predicting grain Cd concentration than other machine learning methods.

### 4.3 How to achieve the 95% goal in the safe use of contaminated agricultural soils?

The Chinese Soil Action Plan set a target of > 95% compliance of contaminant concentrations within food safety limits in contaminated soils by 2030 [61]. Our scenario analyses indicate that raising soil pH to a target pH of 6.5 can reduce the current exceedance of 36.5% to 2.0% (Fig. 4b). Using limestone, this requires 299,000 t CaCO_3_ in total for the study area. Economically, it will cost RMB 1,650 – 4,650 (ca. US $ 220–620) per ha (RMB 119.6 million for the entire study area). This is one of the best cost-effective strategies that should be adopted to help achieve the 95% safe production goal. Lime is widely used as a soil amendment to increase soil pH and reduce grain Cd concentrations [48], [49], [50],[80], [81], [82], [83]. A one-time application of limestone to raise soil pH to 6.5 is highly effective in lowering grain Cd concentration by 70 – 80% to levels below the Chinese food limit, and importantly, the effectiveness can last for at least three seasons without adverse effects on grain yield [50]. More recently, two meta-analysis studies evaluated the effectiveness of liming, and concluded that liming can increase rice yield and reduce grain Cd concentration [48,49]. There is a concern that liming may concomitantly reduce the concentrations of essential elements in rice grain, although this effect was not significant in a field trial [50]. Some micronutrient fertilizers and soil amendments containing Zn and Mn may be used to overcome this problem. It has been shown that one-time amendments of ZnSO_4_ or MnSO_4_ (75 – 150 kg/ha Zn or Mn) were also highly effective in decreasing soil extractable Cd concentrations during soil drainage and the resultant grain Cd accumulation [84]. Finally, another concern regards the durability of the liming effectiveness. Maintaining soil pH around the target pH of 6.5 by applying small amounts of lime every 3-5 years can sustain the beneficial effect.

## 5 Conclusion

In summary, machine learning-based methods can be used to predict rice grain Cd concentrations at a regional scale, with much better performance than the best multiple linear regression method. The most important variables affecting grain Cd accumulation are amorphous Fe-Mn oxides-bound Cd, followed by soil pH, soil moisture content during the late grain filling period, and soil reducible Mn concentration. Scenario analyses with the machine learning-based method reveal that liming the soils in the study area to a target pH of 6.5, together with low-Cd accumulating rice cultivars, can achieve the 95% goal in the safe use of Cd-contaminated soils in the region set by the Chinese Soil Action Plan. This strategy is comparatively easy and cost-effective to implement in Cd-contaminated areas in southern China.

## Declaration of competing interest

The authors declare that they have no conflicts of interest in this work.

## Appendix Supplementary materials

Image, application 1
