# Supplementary material for: Machine learning methods to predict cadmium (Cd) concentration in rice grain and support soil management at a regional scale
Source: Fundam Res. 2023 Mar 10;4(5):1196–205. doi: 10.1016/j.fmre.2023.02.016 (PMC11489518; doi:10.1016/j.fmre.2023.02.016)
Supplement: Supplementary file 1 [file mmc1.docx]

**Supporting Information**

**Machine learning methods to predict cadmium (Cd) concentration in rice grain and support soil management at a regional scale**

Bo-Yang Huang^a,1^, Qi-Xin Lü^a,1^, Zhi-Xian Tang^a^, Zhong Tang^a^, Hong-Ping Chen^a^, Xin-Ping Yang^a^, Fang-Jie Zhao^a^, Peng Wang^a,b,*^

^a^ *Jiangsu Collaborative Innovation Center for Solid Organic Waste Resource Utilization, College of Resources and Environmental Sciences, Nanjing Agricultural University, Nanjing 210095, China*

^b^ *Centre for Agriculture and Health, Academy for Advanced Interdisciplinary Studies, Nanjing Agricultural University, Nanjing 210095, China*

* *Corresponding Author: Peng Wang, E-mail:* [*p.wang3@njau.edu.cn*](mailto:p.wang3@njau.edu.cn)*, Phone: +86 (0)25 8439 5535, Fax: +86 (0)25 8439 5535.*

^1^ *These authors contributed equally to this work.*

Total Pages: 9

Tables: 4

Figures: 5

**Supplementary Tables:**

**Table S1.** The data distribution and statistics of training and validation sets for machine learning models.

|  | **Variables** | **Range** | **Mean** | **Median** | **Variable**  **coefficient** |
| --- | --- | --- | --- | --- | --- |
| Training sets  (n = 280) | C_g-Cd_ (mg/kg) | 0.004 - 1.63 | 0.26 | 0.14 | 1.22 |
|  | C_t-Cd_ (mg/kg) | 0.14 - 3.95 | 0.72 | 0.55 | 0.76 |
|  | C_e-Cd_ (mg/kg) | 0.00 - 0.35 | 0.06 | 0.03 | 1.14 |
|  | C_Fe/Mn-Cd_ (mg/kg) | 0.01 - 1.42 | 0.17 | 0.11 | 1.09 |
|  | C_t-Mn_ (mg/kg) | 90.1 - 2655 | 398.8 | 331.6 | 0.67 |
|  | C_e-Mn_ (mg/kg) | 0.01 - 127.8 | 20.0 | 15.6 | 0.92 |
|  | C_Fe/Mn-Mn_ (mg/kg) | 15.3 - 1457 | 210.7 | 140.5 | 0.94 |
|  | Soil moisture content (%) | 12.0 - 230.3 | 54.4 | 51.2 | 0.44 |
|  | Air-dried soil pH | 4.16 - 7.49 | 5.65 | 5.58 | 0.13 |
| Validation sets  (n = 121) | C_g-Cd_ (mg/kg) | 0.003 - 1.49 | 0.22 | 0.12 | 1.27 |
|  | C_t-Cd_ (mg/kg) | 0.21 - 2.32 | 0.66 | 0.54 | 0.58 |
|  | C_e-Cd_ (mg/kg) | 0.00 - 0.56 | 0.05 | 0.03 | 1.53 |
|  | C_Fe/Mn-Cd_ (mg/kg) | 0.01 - 1.29 | 0.17 | 0.12 | 1.11 |
|  | C_t-Mn_ (mg/kg) | 71.8 - 1892 | 365.6 | 312.7 | 0.63 |
|  | C_e-Mn_ (mg/kg) | 0.01 - 73.8 | 18.7 | 15.7 | 0.84 |
|  | C_Fe/Mn-Mn_ (mg/kg) | 17.2 - 1661 | 182.3 | 129.4 | 1.03 |
|  | Soil moisture content (%) | 13.1 - 186.0 | 56.4 | 54.3 | 0.42 |
|  | Air-dried soil pH | 4.12 - 7.47 | 5.71 | 5.63 | 0.14 |

C_g-Cd_ is the concentration of Cd in rice grain. C_t-Cd_, C_e-Cd_, C_Fe/Mn-Cd_ represents the concentration of total Cd, extracted Cd by CaCl_2_, extracted Cd by NH_4_·HCl in air-dried soil, respectively. C_t-Mn_, C_e-Mn_, C_Fe/Mn-Mn_ represents the concentration of total Mn, extracted Mn by CaCl_2_, extracted Mn by NH_4_·HCl in air-dried soil, respectively.

**Table S2.** The functions and optimized parameters of machine learning models.

| **Method** | **Function** | **Optimized parameters** |
| --- | --- | --- |
| BP neural network  (BP-NN) | neuralnet | hidden = 10 |
|  |  | threshold = 0.05 |
|  |  | learningrate = 0.1 |
|  |  | algorithm = “rprop+” |
|  |  | err.fct = “sse” |
|  |  | act.fct = “logistic” |
| Random forest  (RF) | randomForest | mtry = 3 |
|  |  | ntree = 400 |
| Support vector machines  (SVM) | svm | cost = 100 |
|  |  | gamma = 0.01 |

**Table S3.** Descriptive statistical of soil metal concentrations and soil properties sampled from Xiangtan City, China in 2016, 2019, and 2020.

| **Variables** | **Range** | **Mean** | **Median** | **Variable**  **coefficient** |
| --- | --- | --- | --- | --- |
| Cd (mg/kg) | 0.10 - 5.26 | 0.67 | 0.52 | 0.80 |
| Mn (mg/kg) | 71.8 - 2655 | 388.8 | 323.1 | 0.66 |
| Cu (mg/kg) | 16.7 - 112 | 40.2 | 36.6 | 0.37 |
| Zn (mg/kg) | 54.6 - 956 | 132.4 | 120.5 | 0.46 |
| Soil moisture content (%) | 12.0 - 230 | 55.0 | 51.7 | 0.43 |
| Air-dried soil pH | 4.12 - 7.70 | 5.60 | 5.49 | 0.13 |
| Total organic carbon (g/kg) | 2.74 - 52.0 | 18.9 | 18.3 | 0.34 |

**Table S4.** Linear regression models to predict rice Cd concentrations (n = 401).

| **ID** | **Prediction model** | **R^2^** | ***P*** | **RMSE** |
| --- | --- | --- | --- | --- |
| 1 | ${Log[C}_{g-Cd}]=-0.896+0.182Log[C_{t-Cd}]$ | 0.003 | < 0.001 | 0.586 |
| 2 | ${Log[C}_{g-Cd}]=-0.257+0.413Log[C_{e-Cd}]$ | 0.241 | < 0.001 | 0.511 |
| 3 | ${Log[C}_{g-Cd}]=-0.199+0.779Log[C_{Fe/Mn-Cd}]$ | 0.293 | < 0.001 | 0.494 |
| 4 | ${Log[C}_{g-Cd}]=0.780+0.347\mathrm{Log}\left[ C_{t-Cd} \right]-0.289[\mathrm{pH}_{air-dried}]$ | 0.133 | < 0.001 | 0.546 |
| 5 | ${Log[C}_{g-Cd}]=-0.636+0.487\mathrm{Log}\left[ C_{e-Cd} \right]+0.089[\mathrm{pH}_{air-dried}]$ | 0.244 | < 0.001 | 0.510 |
| 6 | ${Log[C}_{g-Cd}]=1.460+0.802\mathrm{Log}\left[ C_{Fe/Mn-Cd} \right]-0.289[\mathrm{pH}_{air-dried}]$ | 0.428 | < 0.001 | 0.444 |
| 7 | ${Log[C}_{g-Cd}]=2.072+0.401\mathrm{Log}\left[ C_{t-Cd} \right]-0.618\mathrm{Log}\left[ C_{t-Mn} \right]-0.240[\mathrm{pH}_{air-dried}]$ | 0.189 | < 0.001 | 0.529 |
| 8 | ${Log[C}_{g-Cd}]=-0.482+0.578Log\left[ C_{e-Cd} \right]-0.094Log\left[ C_{e-Mn} \right]+0.104[\mathrm{pH}_{air-dried}]$ | 0.251 | < 0.001 | 0.508 |
| 9 | ${Log[C}_{g-Cd}]=2.162+0.836Log\left[ C_{Fe/Mn-Cd} \right]-0.352Log\left[ C_{Fe/Mn-Mn} \right]-0.0.272[\mathrm{pH}_{air-dried}]$ | 0.468 | < 0.001 | 0.428 |
| 10 | ${Log[C}_{g-Cd}]=1.662+0.419Log\left[ C_{t-Cd} \right]-0.070Log\left[ C_{t-Mn} \right]-0.257\left[ \mathrm{pH}_{air-dried} \right]-0.864Log[MC]$ | 0.255 | < 0.001 | 0.507 |
| 11 | ${Log[C}_{g-Cd}]=-0.511+0.487Log\left[ C_{e-Cd} \right]-0.070Log\left[ C_{e-Mn} \right]+0.044\left[ \mathrm{pH}_{air-dried} \right]-0.668Log\left[ \mathrm{MC} \right]$ | 0.288 | < 0.001 | 0.495 |
| 12 | ${Log[C}_{g-Cd}]=1.810+0.863Log\left[ C_{Fe/Mn-Cd} \right]-0.282Log\left[ C_{Fe/Mn-Mn} \right]-0.286\left[ \mathrm{pH}_{air-dried} \right]-1.019Log[MC]$ | 0.563 | < 0.001 | 0.388 |

C_g-Cd_ is the concentration of Cd in rice grain, pH_air-dried_ is the air-dried soil pH, MC is the soil moisture content. C_t-Cd_, C_e-Cd_, C_Fe/Mn-Cd_ represents the concentration of total Cd, extracted Cd by CaCl_2_, extracted Cd by NH_4_·HCl in air-dried soil, respectively. C_t-Mn_, C_e-Mn_, C_Fe/Mn-Mn_ represents the concentration of total Mn, extracted Mn by CaCl_2_, extracted Mn by NH_4_·HCl in air-dried soil, respectively. RMSR is the root mean square error.

**Supplementary Figures:**

**
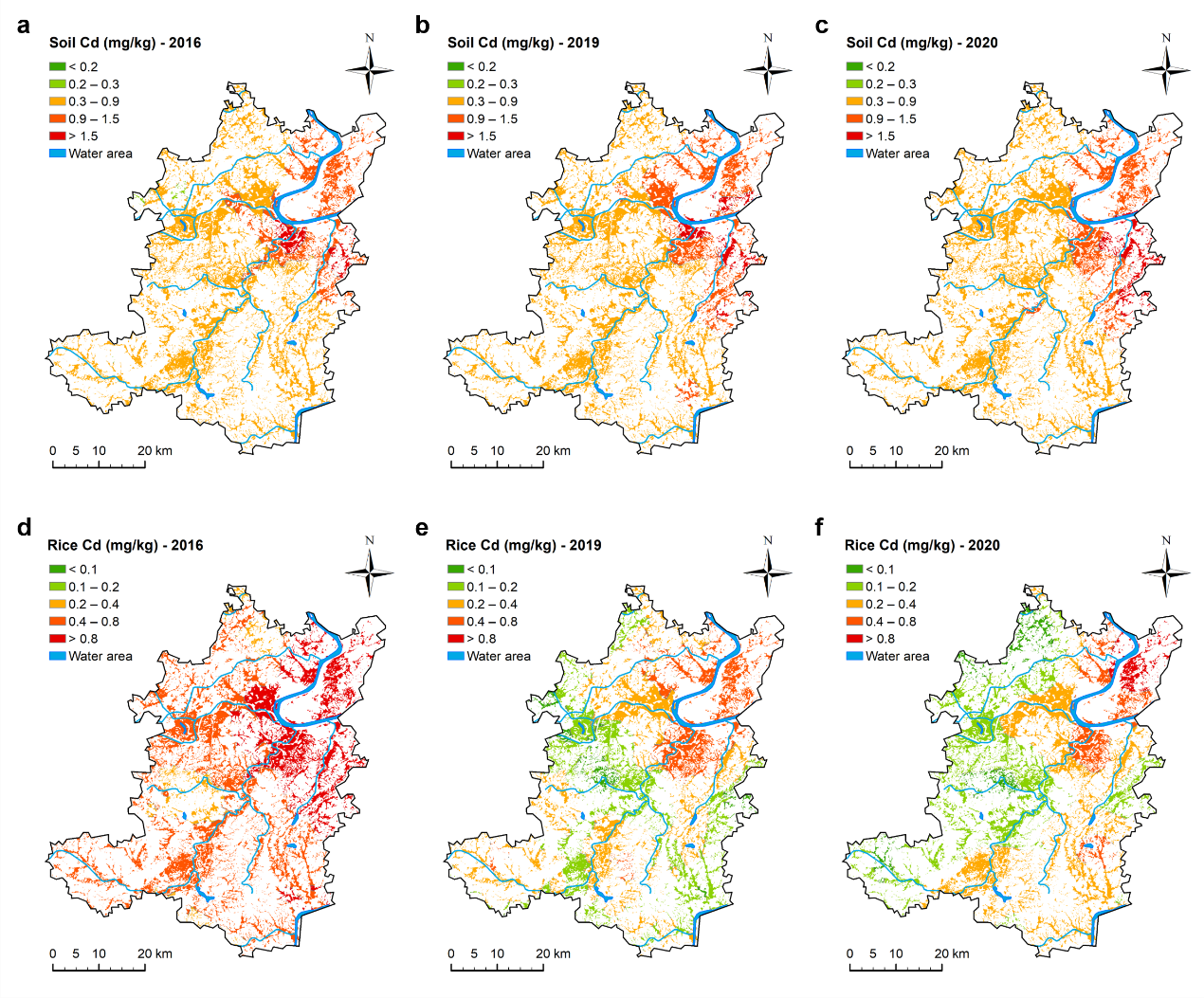
**

**Fig. S1.** Spatial distribution maps of soil and rice grain Cd concentrations in 2016 (a, d), 2019 (b, e), and 2020 (c, f).


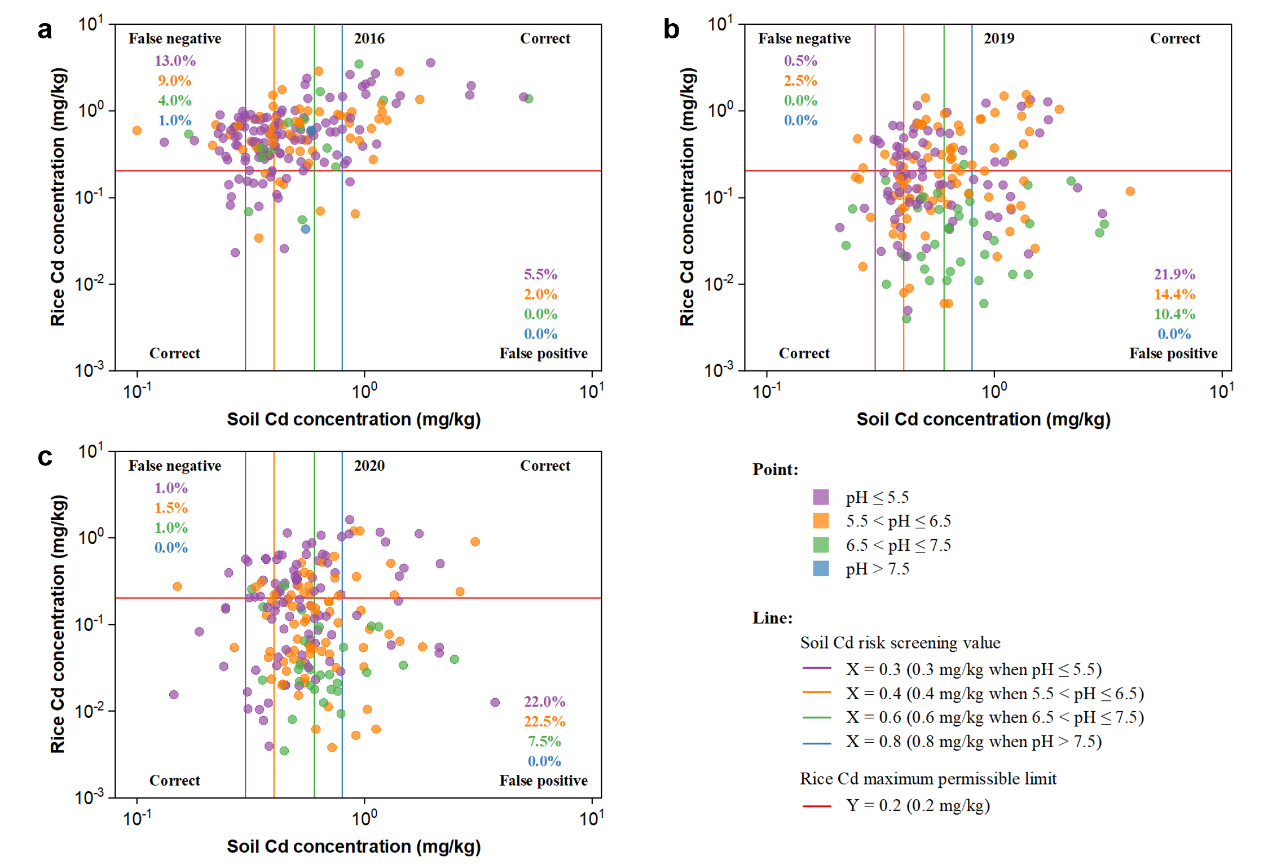


**Fig. S2.** Error rate of using soil Cd to determine whether rice Cd exceeds the standard in 2016 (a), 2019 (b), and 2020 (c).


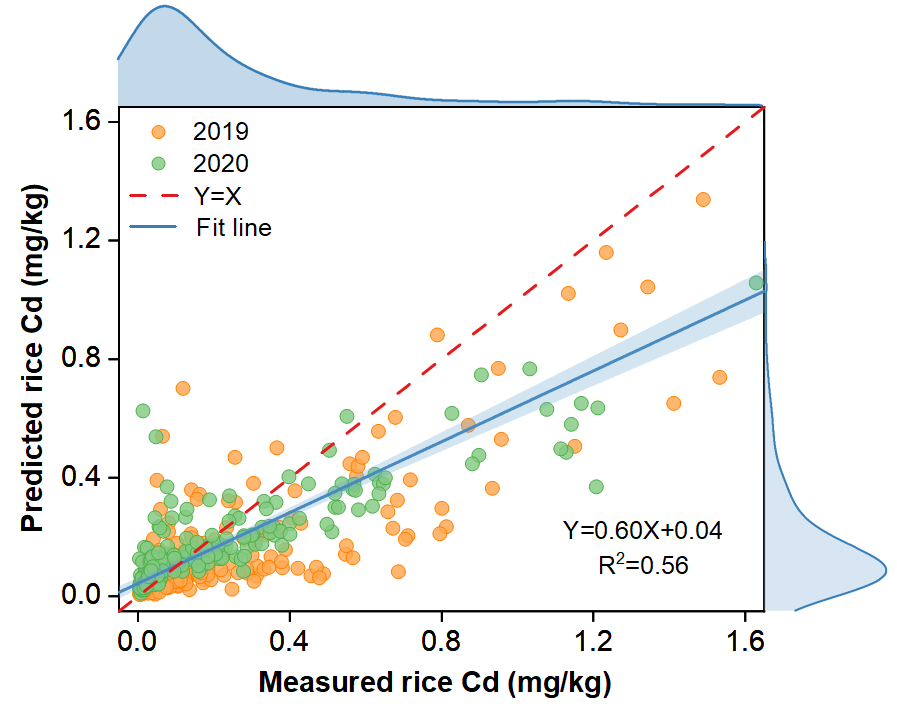


**Fig. S3.** Scatter plots of measured rice grain Cd concentration versus predicted rice grain Cd concentration values (n = 401) of 2019 and 2020 based on stepwise multiple linear regression.

**
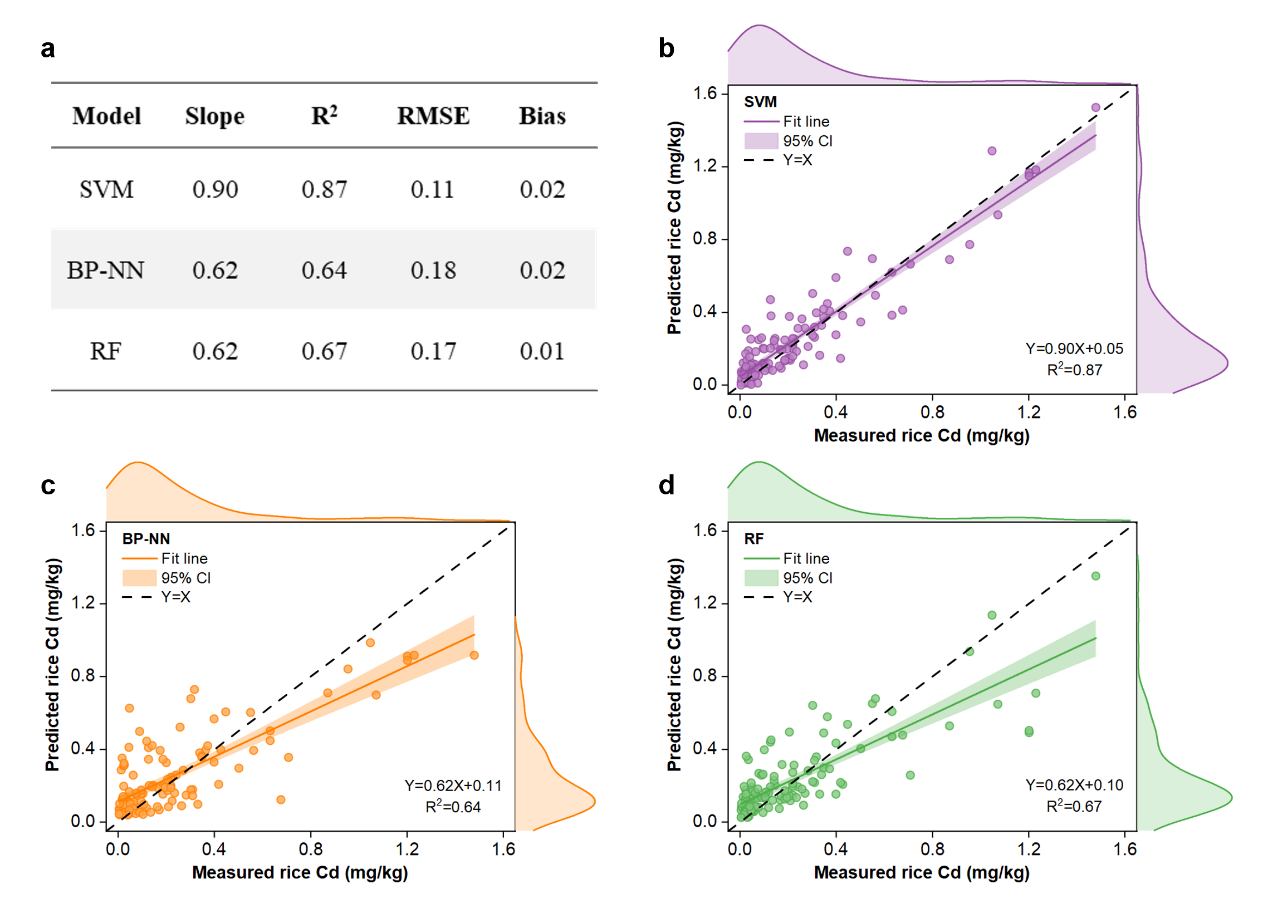
**

**Fig. S4.** Comparison of three machine learning models. (a) Prediction error indices of models. Scatter plots of measured concentration versus predicted concentration values in the validation dataset based on the BP-NN (b), PF (c), and SVM (d) models (n = 121). RMSR is the root mean square error. SVM, BP-NN, RF represents the support vector machines, BP neural network, and random forest, respectively.

**
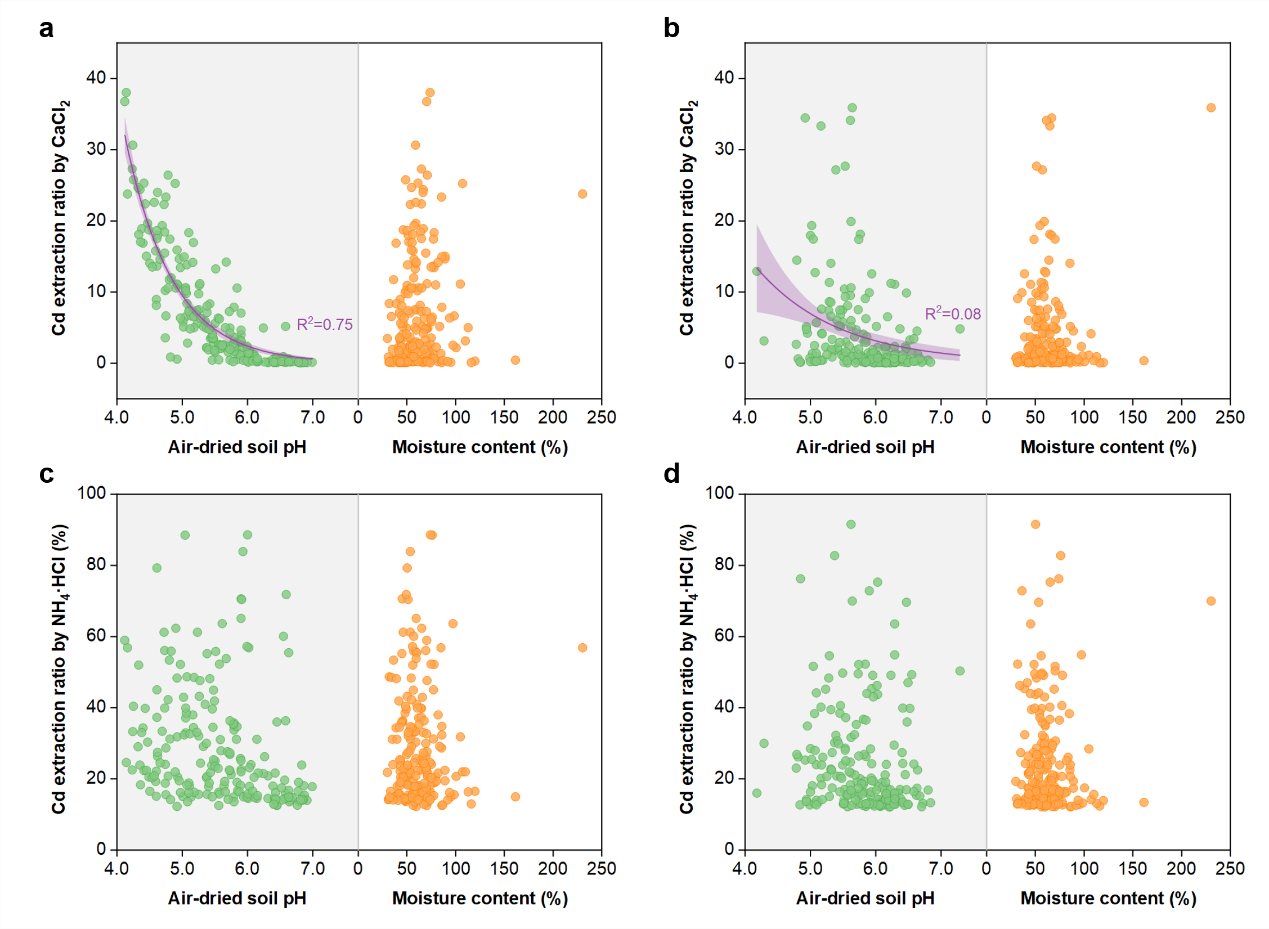
**

**Fig. S5.** The pairing relationship between Cd extraction ratio by CaCl_2_ and two properties (soil pH, soil moisture content) for air-dried (a) and field-moist soil (b) in 2020. The pairing relationship between Cd extraction ratio by NH_4_·HCl and two properties (pH, moisture content) for air-dried (c) and field-moist soils (d) in 2020.
